# Heterogeneity of Organization of Subcompartments in DSB Repair Foci

**DOI:** 10.3389/fgene.2022.887088

**Published:** 2022-07-18

**Authors:** Natnael G. Abate, Michael J. Hendzel

**Affiliations:** Departments of Oncology and Cell Biology, Faculty of Medicine and Dentistry, University of Alberta, Edmonton, AB, Canada

**Keywords:** DNA double-stand break, fluorescence microscopy, homologous recombination (HR) pathway, nonhomologous end-joining (NHEJ), DNA repair, cell nucleus, nuclear compartmentalization

## Abstract

Cells assemble compartments around DNA double-strand breaks (DSBs). The assembly of this compartment is dependent on the phosphorylation of histone H2AX, the binding of MDC1 to phosphorylated H2AX, and the assembly of downstream signaling and repair components. The decision on whether to use homologous recombination or nonhomologous end-joining repair depends on competition between 53BP1 and BRCA1. A major point of control appears to be DNA replication and associated changes in the epigenetic state. This includes dilution of histone H4 dimethylation and an increase in acetylation of lysine residues on H2A and H4 that impair 53BP1 binding. In this article, we examined more closely the spatial relationship between 53BP1 and BRCA1 within the cell cycle. We find that 53BP1 can associate with early S-phase replicated chromatin and that the relative concentration of BRCA1 in DSB-associated compartments correlates with increased BRCA1 nuclear abundance as cells progress into and through S phase. In most cases during S phase, both BRCA1 and 53BP1 are recruited to these compartments. This occurs for both IR-induced DSBs and breaks targeted to an integrated LacO array through a LacI-Fok1-mCherry fusion protein. Having established that the array system replicates this heterogeneity, we further examined the spatial relationship between DNA repair components. This enabled us to precisely locate the DNA containing the break and map other proteins relative to that DNA. We find evidence for at least three subcompartments. The damaged DNA, single-stranded DNA generated from end resection of the array, and nuclease CtIP all localized to the center of the compartment. BRCA1 and 53BP1 largely occupied discrete regions of the focus. One of BRCA1 or 53BP1 overlaps with the array, while the other is more peripherally located. The array-overlapping protein occupied a larger volume than the array, CtIP, or single-stranded DNA (ssDNA). Rad51 often occupied a much larger volume than the array itself and was sometimes observed to be depleted in the array volume where the ssDNA exclusively localizes. These results highlight the complexity of molecular compartmentalization within DSB repair compartments.

## Introduction

The preservation of genetic information is critical for cell and species survival. DNA double-strand breaks (DSBs) can compromise the integrity of this genetic information. Consequently, cells have evolved a complex DNA damage response that senses damage and orchestrates the proper repair and maintenance of genetic sequence. Upon DSB formation, the cell organizes up to 1.5 million base pairs surrounding the DSB into a nuclear compartment characterized by a histone mark, phosphorylated serine 139 of histone H2AX (γH2AX) (Iacovoni et al., 2010; Caron et al., 2012; Aymard et al., 2014; Aymard and Legube, 2016). This compartment acts as a repair site and source of signaling for cell cycle arrest until the DSB is repaired (Jackson, 2002; Bekker-Jensen and Mailand, 2010; Hustedt and Durocher, 2017). The compartment is initiated by the recruitment of the MRN complex (MRE11, RAD50, and NBS1) to the break (Lavin, 2004; Lee and Paull, 2005). The MRN complex can recognize the DSB and activate Ataxia-telangiectasia-mutated (ATM) kinase, which will phosphorylate histone H2AX at serine 139 to generate γH2AX (Stucki and Jackson, 2004; Bekker-Jensen et al., 2005). ATM kinase can also phosphorylate mediator of DNA damage checkpoint (MDC1), forming a complex with γH2AX to recruit E3 ligase ring finger 8 (RNF8) (Huen et al., 2007; Kolas et al., 2007; Mailand et al., 2007). Ubiquitylation mediated through RNF8 recruits E3 ligase ring finger 168 (RNF168) (Kolas et al., 2007; Mailand et al., 2007; Doil et al., 2009; Stewart et al., 2009). RNF168-mediated ubiquitylation that occurs on histone H2A K13/15 is directly recognized by 53BP1 at DNA DSB, while polyubiquitylation by RNF8/UBC13 generates K63-linked ubiquitin chains that can bind BRCA1 A complex through ubiquitin-interacting motifs in RAP80 (Kolas et al., 2007; Mailand et al., 2007; Sobhian et al., 2007; Wang et al., 2007; Mattiroli et al., 2012; Fradet-Turcotte et al., 2013).

Among the epigenetic changes that regulate the repair pathway, those that impact the competition between 53BP1 and BRCA1 and the downstream effectors are of particular interest because they dictate the repair outcome. Upon recruitment of 53BP1 to DNA DSB sites, 53BP1 can recruit other effector proteins such as RIF1 and PTIP1 (Gong et al., 2009; Zimmermann et al., 2013), while BRCA1 can form a complex with CtIP and MRN to promote 5′–3′ end resection and recruit PALB2/BRCA2 complex to promote Rad51 loading onto the 3′ (Chen et al., 2008; Escribano-Díaz et al., 2013; Simonetta et al., 2018; Krais et al., 2021). 53BP1 and BRCA1 compete to determine the DSB repair pathway choice (Bouwman et al., 2010; Bunting et al., 2010). This may be reflected in their spatial organization within DSB-associated compartments. BRCA1 is proposed to displace 53BP1 from chromatin near the DSB, consistent with super-resolution fluorescence microscopy experiments revealing peripheral localization of 53BP1 accompanied by accumulation of BRCA1 toward the interior of the compartment (Chapman et al., 2012; Feng et al., 2015). However, transmission electron microscopy revealed a peripheral localization of chromatin in DSB-associated compartments (Strickfaden et al., 2015). This suggested that repair might take place on the periphery of the compartment, and its central domain may function in sequestering molecules away from the break.

To better understand how the organization of repair proteins within the DSB-associated compartment relates to DSB repair pathway choice, we need to know the location of the DNA containing the break. At present, visualizing γH2AX using specific antibodies is the best method to identify DNA DSB sites. However, chromatin immunoprecipitation experiments have demonstrated that histones and γH2AX may be displaced from the actual site of the break (Arnould et al., 2021), and consequently, we cannot determine the exact position of the DNA break using γH2AX. This complicates interpreting the relationship between how molecules are organized within the repair compartment and how this organization relates to function. This uncertainty is increased if liquid compartments are forming in association with the break.

Liquid–liquid unmixing and phase separation are emerging mechanisms of generating membraneless compartments within the nucleus (Razin and Gavrilov, 2020; Nesterov et al., 2021). Poly(ADP-ribose) can initiate phase separation at DNA damage sites and plays an important role in regulating phase separation in the cytoplasm (Altmeyer et al., 2015; Rack et al., 2021). Similarly, both RNA and 53BP1 have been proposed to initiate phase separation within DSB-associated compartments (Kilic et al., 2019; Pessina et al., 2019; Guo et al., 2021; Zhang et al., 2022). In this light, it is important to appreciate that the sites of steady-state accumulation of these proteins may reflect their preferred partitioning into a distinct solvent microenvironment and may not reflect the sites of action on the broken DNA or association with chromatin. In other words, differences in localization may not simply reflect differences in distribution along the chromatin fiber. Thus, it is critical to know the location of the break site(s) within the compartment. This is possible using a model DSB system where integrated arrays of the Lac operon sequence are inserted into the genome and specifically targeted by a fusion protein of the LacI DNA-binding domain and the Fok1 endonuclease domain. The incorporation of a fluorescent protein tag on this fusion protein enables the direct visualization of the break site, and the organization of DNA damage response proteins can be studied in relation to DSB.

In this study, we demonstrated that there are multiple classes of DSB repair compartments based on BRCA1 and 53BP1 abundance and organization. These morphological classifications correlate well with cell cycle progression-associated changes in 53BP1 foci abundance reported previously (Chapman et al., 2013; Escribano-Díaz et al., 2013; Feng et al., 2015; Michelena et al., 2021; Swift et al., 2021). This might be explained by epigenetic changes accompanying the replication of chromatin. However, in contrast to our expectations, we found that 53BP1 can colocalize with newly replicated DNA following ionizing radiation treatment. There is an ongoing increase in 53BP1 nuclear concentration throughout the cell cycle, while BRCA1 increases rapidly at the onset of the S phase. Typically, both proteins were present in individual foci, but the relative abundance in foci correlated with BRCA1 expression, rather than 53BP1, and total BRCA1 nuclear abundance until late S phase, where 53BP1 formed few foci and had a more prominent nuclear staining outside of foci. After demonstrating the conservation of DSB compartment heterogeneity in the model Lac array DSB system, we examined the relationship between 53BP1, BRCA1, and downstream effectors relative to the location of the DSB (Tang et al., 2013; Arnould et al., 2021). This array system contains 265 tandem repeat LacI binding sites where DSBs can be generated by a LacI–Fok1 fusion protein that is further tagged with mCherry to enable visualization of the array. This allows unambiguous positioning of the damaged DNA. We found that the damaged DNA is located centrally and is the compartment enriched in ssDNA and DNA end resection factors. In contrast, NHEJ and HR factors exist in larger volumes that vary in their spatial relationship with the array. Deconvolution of confocal images suggests that there are at least three subcompartments in the DSB repair compartment—the DNA containing the break, biomolecules associated with 53BP1, and biomolecules associated with BRCA1. While either 53BP1 or BRCA1, but not both, can be found on arrays in individual cells, these compartments, unlike ssDNA and CtIP, extend beyond the dimensions of the array and are further surrounded by the complementary BRCA1-rich or 53BP1-rich compartment. Moreover, since cells containing more centralized 53BP1 have lower DNA content than those with centralized BRCA1, BRCA1 displacement of 53BP1 from the center of the focus may depend on S-phase progression. Since DNA is found in all three compartments, subcompartments could arise through decorating the chromatin fiber or through liquid–liquid unmixing into separate compartments through phase separation.

## Materials and Methods

### Cell Culture

WT U2OS and U2OS expressing the Lac array were maintained in Dulbecco’s modified Eagle medium (DMEM) with 10% FBS and 1% penicillin–streptomycin at 37°C. All cells were maintained in sterile cell culture and tested for mycoplasma.

### Immunofluorescence

Cells were grown on a glass coverslip in a 35-mm tissue culture dish. DSB formation was initiated and then cells were fixed 1 h later with 4% paraformaldehyde for at least 10 min at room temperature. Following fixation, the fixative was removed and 1–2 ml of 1× PBS was added. PBS was removed and cells were permeabilized by adding 1–2 ml of PBS 0.5% Triton X-100 for at least 5 min. Cells were rinsed two times with 1× PBS and left in 1× PBS. Cells were incubated with a primary antibody by placing the coverslip cell side down on a 30-µl drop of antibody on Parafilm, avoiding air bubbles, for 45 min. The cover of a 35-mm dish was left on top to minimize dehydration. Cells were rinsed once with 1× PBS with 0.1% Triton X-100 to permeabilize the membrane of the cells and then rinsed again with 1× PBS and left in 1× PBS. Cells were incubated with a secondary antibody by placing the coverslip cell side down on a 30-µl drop of antibody on a Parafilm for 45 min. After 45 min, cells were rinsed once with 1× PBS with 0.1% Triton X-100 and twice with 1× PBS. Coverslips were then mounted cell side down onto slides with mounting media (20 µl; 90% glycerol, 10% PBS, 0.1% *p*-phenylenediamine, and multichannel TetraSpeck microspheres) per coverslip.

### Initiating DNA Double-Strand Breaks

U2OS 265 cells were gifted from the Roger Greenberg’s laboratory (Tang et al., 2013). Cells were grown on a glass coverslip and treated with 0.5 mM Shield1 and 10 mM 4-OHT for 1 h before fixation with 4% paraformaldehyde (Shield1 [632189], Takara; 4-OHT [68047-06–03], Sigma-Aldrich). Cells were washed with PBS and permeabilized with 0.5% Triton X-100 in PBS for 5 min and incubated with primary antibody for 45 min and washed with PBS. Then, incubated with secondary antibody for another 45 min and washed with PBS. Coverslips were mounted on slides using mounting media (90% glycerol, 10% PBS, and 0.1% *p*-phenylenediamine).

### Antibodies, Chemicals, and Reagents

Conjugated 53BP1 rabbit polyclonal antibody was obtained from Novus (NB100-309AF488); BRCA1 mouse monoclonal antibody (5C-6934) from Santa Cruz; BRCA1 rabbit polyclonal antibody (07-434) and γH2AX mouse monoclonal antibody (2535291) from Millipore; rabbit polyclonal antibody (39117) from Active Motif; RAD51 rabbit polyclonal antibody (20-001) from Bio Academia; CtIP mouse monoclonal antibody (61141) and RPA rabbit polyclonal antibody (AB76420) from Abcam; RAP80 rabbit polyclonal antibody (14466), RIF1 rabbit polyclonal antibody (A300-569A), mouse monoclonal antibody (200-301-H50), and BrdU mouse monoclonal antibody (B5002) from Rockland; EdU Click-iT (C10338) from Sigma-Aldrich; Alexa 488 goat anti-mouse antibody (A11OC1) from Molecular Probes; Cy5 goat anti-mouse antibody (195-175-166), anti-rabbit antibody (111-175-144), and Cy3 goat anti-mouse antibody (115-165-146) from Jackson.

### BrdU-ssDNA

U2OS 265 cells were preincubated with 10 µM BrdU for 18 h, followed by 1-h incubation with Shield1 and 4-OHT. Cells were fixed with 4% paraformaldehyde for 10 min at room temperature. Cells were washed with PBS and permeabilized with 0.5% Triton X-100 in PBS for 5 min and incubated with anti-mouse BrdU antibody (B5002) overnight.

### EdU Pulse Labeling

U2OS WT cells were grown on a glass coverslip in a 35-mm tissue culture plate. Cells were treated with 10 µM 5′-ethynyl-2-deoxyuridine (EdU) for 30 min and 6 h, irradiated with 2 Gy, and then fixed after 1 h with 4% paraformaldehyde. Cells were washed with PBS and permeabilized with 0.5% Triton X-100 in PBS for 5 min. Cells were again washed with PBS and incubated with EdU Click-IT reaction (Imaging Kit, Invitrogen) using Alexa 488 Azide dye for 1 h to label the newly replicated chromatin.

### Image Acquisition and Quantification

Images were captured using a Leica SP8 laser scanning confocal microscope (100× 1.4 N.A. oil immersion objective). Tetra beads were added for image corrections and to assess and correct channel alignment. Between 5 and 10 z-plane images were acquired with 200–400 nm step size using a 405-nm laser for DAPI and a white light laser for Alexa 488, Cy3, mCherry, and Cy5. To excite DAPI, 405 nm laser was used, 488 nm excitation was used for Alexa 488-labeled antibodies, 594 nm excitation for mCherry, 561 nm excitation for Cy3, and 649 nm excitation for Cy5. Images were analyzed using Bitplane Imaris and ImageJ software. DAPI intensity was used to quantify DNA and identify the cell cycle position and observe the relative difference between 53BP1 and BRCA1. Quantification of images was done post-baseline subtraction to remove any background signal. Maximum intensity projection images were used to generate the summed nuclear intensities. Radial profile plots were obtained using ImageJ in which an area was selected and a radial increase of 75 nm per pixel.

## Results

### Heterogeneity in 53BP1 and BRCA1 Recruitment

**FIGURE 1:**
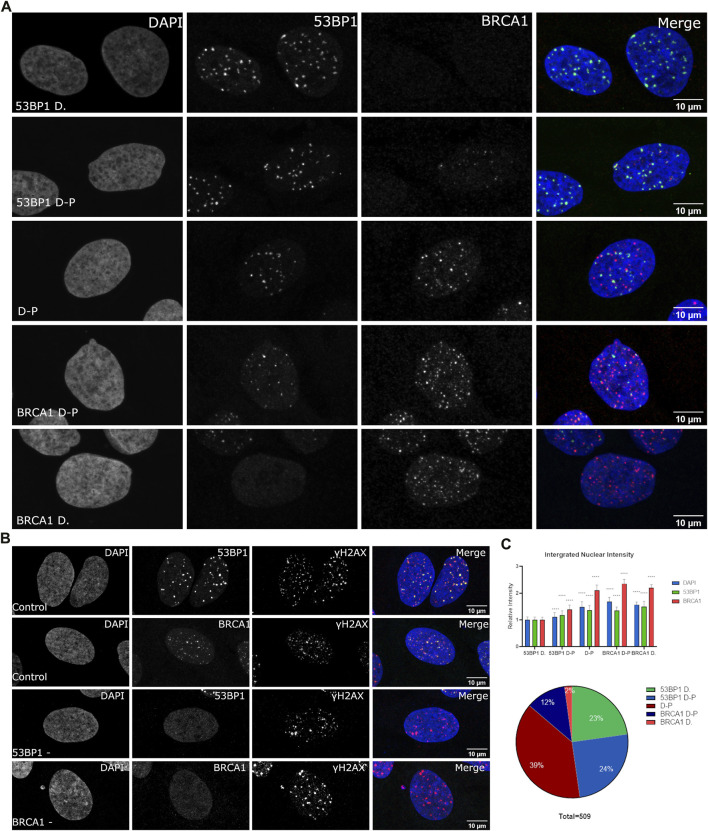
Variation in BRCA1 and 53BP1 recruitment in asynchronous cell populations. WT U2OS cells were fixed 1 h post 2 Gy irradiation and immunostained with antibodies for 53BP1, BRCA1, and γH2AX showing heterogeneity in recruitment to DSB. **(A)** Cells were classified subjectively into five categories based on their relative abundance of BRCA1 and 53BP1 in foci: 53BP1-dominant cell (53BP1 D), 53BP1-dominant double-positive cell (53BP1 D–P), 53BP1–BRCA1–positive cell (D–P), BRCA1 dominant double-positive cell (BRCA1 D–P), and BRCA1-dominant cell (BRCA1 D). Cells were normalized using the intensity values of the 53BP1-dominant category, where cells predominately are in G1 with low DAPI intensity. **(B)** DNA double-strand breaks were confirmed in cells that were negative for 53BP1 foci and cells that were negative for BRCA1 foci using γH2AX as a marker for DSB foci. **(C)** The DNA, BRCA1, and 53BP1 were measured for 509 cells obtained from four separate experiments. These were then plotted for the total nuclear content using the integrated nuclear intensity of each normalized to the 53BP1-dominant category. The proportion of cells in each category is also shown. Quantification of different categories. Error bars represent mean ± SD, ns represents nonsignificant (*p* ≥ 0.01), *p ≤ 0.1, **p ≤ 0.01, ***p ≤ 0.001, ****p ≤ 0.0001 obtained from pair-wise comparisons of each value relative to the 53BP1-dominant category using a Student’s *t* test. The scale bar represents 10 µm.

53BP1 and BRCA1 play a critical role in the cell cycle-dependent regulation of DNA repair. 53BP1 is often used interchangeably with histone H2AX phosphorylation to enumerate DSBs despite cell cycle-dependent relationships on 53BP1 foci abundance being reported (Escribano-Díaz et al., 2013; Feng et al., 2015; Michelena et al., 2021; Swift et al., 2021). We sought to assess the heterogeneity in their association with sites of DNA DSBs within asynchronous cell populations. We conducted immunofluorescence using wild type U2OS cells and specific antibodies directed against 53BP1 and BRCA1. We treated cells with 2Gy radiation, fixed 1 h post-treatment, and determined differences in DSB-associated compartments between individual cells. We classified individual cells based on their apparent dominance of 53BP1 versus BRCA1 in merged datasets and then quantified the relative nuclear content of BRCA1, 53BP1, and DNA (Figure 1A). To obtain the nuclear DNA content, we measured the integrated nuclear intensity of DAPI, 53BP1, and BRCA1. To confirm the presence of DSBs in cells that contained either no 53BP1 or no BRCA1 foci, cells were costained for phosphorylated H2AX. These cells show abundant DSBs despite the failure to recruit one of 53BP1 or BRCA1 (Figure 1B). Cells that strongly recruit 53BP1 but have very little or no BRCA1 in foci were found to have the lowest DNA content and low BRCA1 total nuclear intensity (Figure 1C). The progression toward BRCA1 dominance correlates with increased BRCA1 total nuclear intensity and DNA content. 53BP1 concentration also increases with DNA content but is abundant in all categories. These results demonstrate the considerable heterogeneity of DSB foci composition in both individual cells and in a population and, in general, correlate well with the reported loss of 53BP1 during the progression into S phase (Escribano-Díaz et al., 2013; Feng et al., 2015). Note that the BRCA1-dominant double-positive (BRCA1 D-P) category had slightly higher BRCA1 content and DNA content than the BRCA1-dominant category. This suggests that this arises later in the cell cycle than in the BRCA1-dominant category and is consistent with the recovery of 53BP1 binding in G2 (Simonetta et al., 2018). Other features of this subset are the presence of BRCA1 single-positive foci and a more apparent nucleoplasmic signal for 53BP1 relative to the G1- and early S-phase cells.

### Changes in DSB Repair Focus Composition During S-Phase Progression

**FIGURE 2:**
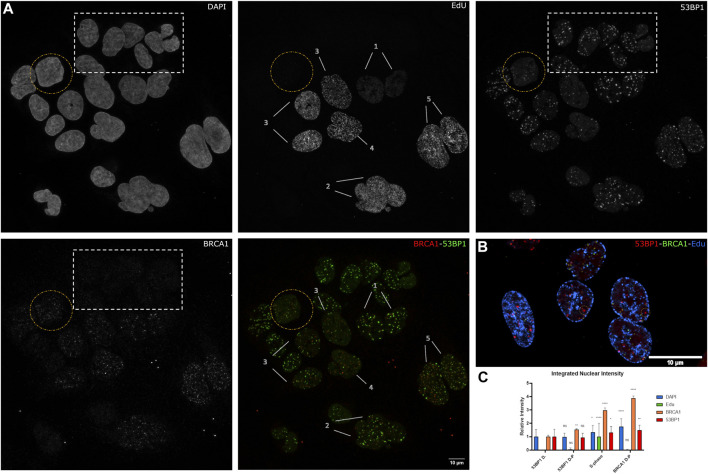
The relationship between S-phase progression, BRCA1, and 53BP1. **(A)** U2OS cells were pulse labeled with EdU 30 min before 2 Gy irradiation. Cells were fixed 1 h postirradiation and immunostained with Click-IT reaction, 53PB1, and BRCA1. **(1–5)** indicate increasing progression through S phase. The yellow circle highlights a BRCA1-dominant non-S-phase cell. The white box indicates 53BP1-dominant non-S-phase cells and **(1–5)** indicate progression through S phase with **1** being the earliest stage and **5** being the latest stage. **(B)** Examples of late S-phase cells showing 53BP1-rich foci. **(C)** Quantification of BRCA1, 53BP1, and DNA intensities with S phase categorized as one and normalized to 53BP1-dominant category. Error bars represent mean ± SD, ns represents nonsignificant (*p* ≥ 0.01), *p ≤ 0.1, **p ≤ 0.01, ***p ≤ 0.001, ****p ≤ 0.0001 obtained from pair-wise comparisons of each value relative to the 53BP1-dominant category using a Student’s *t* test. Note that multiwavelength fluorescent TetraSpeck microspheres were added for alignment corrections. These are present as small fluorescent dots outside of the cells in all channels. The scale bar represents 10 µm.

To better understand the transition from 53BP1-dominated foci to BRCA1-dominated foci in relation to the cell cycle, we pulse-labeled cells with 5′-ethinyl-2′deoxyurdine (EdU) for 30 min prior to irradiation and then fixed cells 1 h after irradiation. This enables the differences in BRCA1 and 53BP1 content to be characterized relative to progression through S phase (Figure 2A,B). Notably, 53BP1-dominant and 53BP1-dominant double-positive (53BP1 D-P) phenotypes both do not incorporate EdU and differ primarily in BRCA1 total nuclear concentration (Figure 2C). In the 53BP1-dominant category, BRCA1 generates very weak nuclear staining and is difficult to detect. Consistent with these cells being pre-replicative, they have the lowest amount of DNA and are not distinguishable based on DNA content (Figure 2C). The early S-phase cells show label incorporation broadly throughout the interior of the nucleus (Figure 2A, patterns 1,2), while this labeling pattern gets increasingly coarse as cells progress through S phase (Figure 2A, patterns 3–5, Figure 2B). The late S-phase cells are easily identified based on the replication of heterochromatin being visible as comparatively large domains of incorporation, often in perinuclear or perinucleolar regions (Figure 2B). Note that in these cells, we can assume that most of the remaining chromatin has been replicated. These cells are notable for their reduction in the number of 53BP1-positive foci and a more diffuse nuclear 53BP1 signal outside of DSB sites (Figure 2A). However, even in these cells, there are consistently examples of foci that are strongly biased toward 53BP1 (circles in Figure 2B). In S-phase cells, there are BRCA1-positive, 53BP1-negative/low foci, foci that are double-positive, and 53BP1-positive, BRCA1-negative/low foci. The final class of cells is positive for both, but negative for EdU incorporation (circled in Figure 2A). These correspond to the BRCA1 D-P phenotype in Figure 1 and reflect G2 cells based on their DNA content.

**FIGURE 3:**
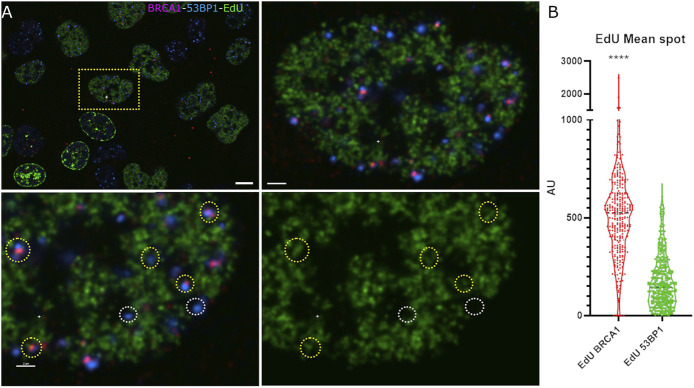
53BP1-rich foci are found in association with newly replicated chromatin in early S phase. **(A)** U2OS cells pulse labeled with EdU and irradiated were examined for newly replicated chromatin distribution (EdU, green), 53BP1 (blue), and BRCA1 (red). An early S-phase cell is highlighted in the upper left panel showing the field of labeled cells. The top right panel shows a higher magnification view of the same cell. The bottom two panels show a subregion of this nucleus with (left) and without (right) the 53BP1 and BRCA1 channels. The yellow circles highlight chromatin that is positive for 53BP1. These same foci show varying amounts of BRCA1. The white circles highlight 53BP1 foci that clearly reside in regions that have not been replicated. **(B)** Violin plot showing the EdU mean intensity in BRCA1 versus 53BP1 foci in early S-phase cells where EdU is indicative of DNA replication. Quantification of EdU BRCA1 mean intensity versus EdU 53BP1 mean intensity, ****p ≤ 0.0001. The scale bar represents 2 µm.

A loss of 53BP1 foci has been previously associated with progression through S phase (Pei et al., 2011; Saredi et al., 2016). A number of epigenetic mechanisms associated with DNA replication, including dilution of histone H4 lysine 20 methylation as newly synthesized histones are deposited, the Tip60-dependent acetylation of histone H2A on lysines 13 and 15, and the MOF-dependent acetylation of H4 lysine 16 (Akhtar and Becker, 2000; Li et al., 2010; Jacquet et al., 2016). Consistent with this, Pellegrino et al. (2017) examined the distribution of 53BP1 foci relative to EdU incorporated into newly synthesized DNA and found that 53BP1 foci did not colocalize with newly replicated chromatin. Thus, we would predict that in early S phase, where 53BP1 foci are prominent, they will localize to unreplicated DNA, and the replicated chromatin will be refractory to 53BP1 assembly. Although this epigenetic change has been shown to reduce 53BP1 occupancy in the presence or absence of BRCA1, these epigenetic differences may not be sufficient to prevent the binding of 53BP1 to newly replicated DNA in S phase (Michelena et al., 2021). Figure 3A shows the relationship between EdU incorporation, BRCA1, and 53BP1 in an early S-phase nucleus. Examples of EdU-labeled chromatin that are double positive for BRCA1 and 53BP1 are highlighted with yellow circles. Examples of 53BP1 located on unreplicated chromatin are illustrated with white circles. We analyzed EdU incorporation at the centers of 53BP1 intensity versus centers of BRCA1 intensity. These results show considerable overlap in the range of EdU concentrations associated with BRCA1 and 53BP1. While there is a tendency for BRCA1 foci to be more closely associated with sites of EdU incorporation, 53BP1 can associate with replicated chromatin and BRCA1 is found in unlabelled regions. Rather than being determined by the underlying chromatin state, the abundance of BRCA1-rich foci correlates more strongly with BRCA1 abundance (Figure 3, see also Figures 1C, 2A,C note the concordant increase in 53BP1 concentration outside of foci). To better understand how BRCA1 total nuclear concentration changes during S-phase progression, we pulsed cells for 6 h in the absence of DNA damage, divided cells into fully labeled, partially labeled early in S phase, and partially labeled late (late S-phase staining pattern with reduced EdU incorporation). Plotting the integrated nuclear intensity revealed a rapid increase in BRCA1 content as labeling increased in the partially labeled early S-phase cells. This suggests that limited BRCA1 concentrations may influence focus composition in early S phase in addition to changes in epigenetic state that favor BRCA1 binding over 53BP1.

### Spatial Relationships of 53BP1 and BRCA1 to the DSB Site

**FIGURE 4:**
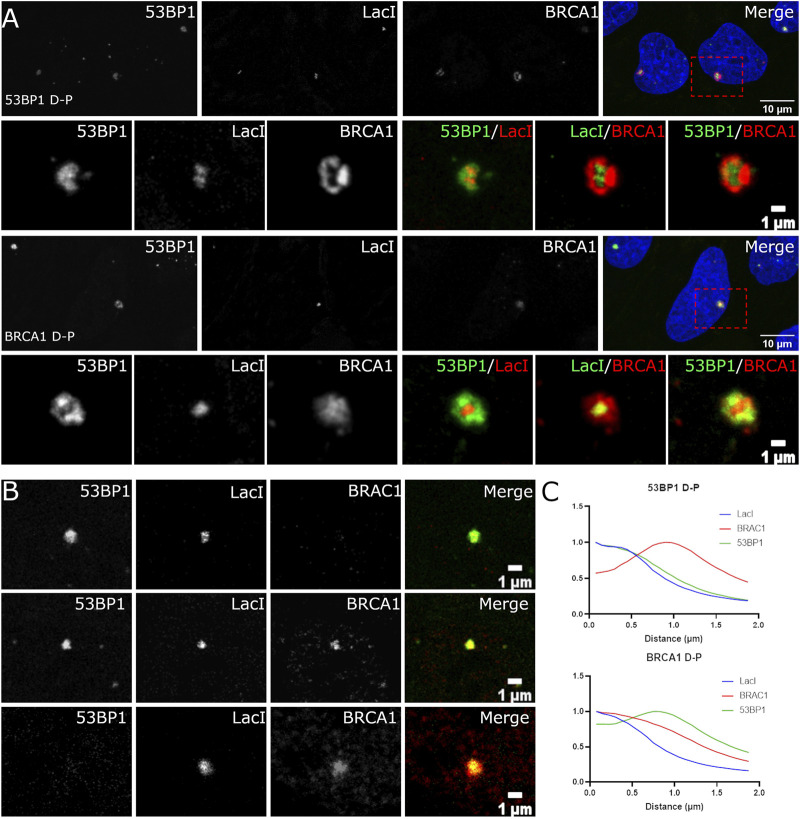
Localization of BRCA1 and 53BP1 relative to the site of the damaged DNA. **(A)** U2OS 265 cells were fixed 1 h post-treatment with 4-OHT and Shield1 to induce DSB formation and then immune stained with 53BP1 and BRCA1. Examples of 53BP1- and BRCA1-dominant double-positive foci are shown. The panel on the far right in rows 1 and 3 show the nucleus with DAPI in blue, BRCA1 in red, and 53BP1 in green. The highlighted region is shown enlarged in the corresponding panels underneath. **(B)** The foci were categorized based on the relative difference in DSB occupancy: 53BP1-dominant cell (53BP1 D), 53BP1-dominant double-positive cell (53BP1 D-P), 53BP1–BRCA1-positive cell (D-P), BRCA1-dominant double-positive cell (BRCA1 D-P), and BRCA1-dominant cell (BRCA1 D). **(C)** Radial profile plots of 30 DSB over three experiments reveal three overlapping distributions that differ in the centralization of BRCA1 or 53BP1. The scale bar represents 10 µm for images illustrating the nucleus (top panel) and scale bar represents 1 µm for individual breaks.

Super-resolution studies of DNA DSBs reveal that individual classes of proteins are not homogeneously distributed throughout the focus (Reindl et al., 2017, 2022; Schwarz et al., 2019; Michelena et al., 2021). For example, BRCA1 and 53BP1 occupy distinct regions of the compartment (Chapman et al., 2012; Mok and Henderson, 2012; Schwarz et al., 2019). Understanding these relationships is complicated by cell cycle-dependent differences in BRCA1 and 53BP1 spatial organization in foci. It is further complicated by the lack of knowledge of where the broken DNA resides within the focus. We sought to examine the spatial organization of individual DSB compartments by exploiting a system where an array of Lac repressor DNA-binding sequences are inserted into U2OS cells. An inducible and rapidly degradable fusion protein containing the LacI DNA-binding domain fused to the Fok1 nuclease domain and mCherry allows us to rapidly induce DSBs and identify the location of the break sites within the assembled focus. We first confirmed that the same distributions of 53BP1 and BRCA1 could be observed in the array system as we observed for IR-induced DSBs. Figure 4A shows that the induction of the nuclease results in the labeling of a single spot within the nucleus that enriches the LacI fusion protein. We found evidence for the same classes of foci as we observed in the asynchronous cell population. Both BRCA1 D-P and 53BP1 D-P foci were observed with the dominant protein localizing more centrally. Notably, both BRCA1 and 53BP1 show localization that overlaps with and extends beyond the array. This can be seen in the radial profile distribution of BRCA1-dominant and 53BP1-dominant foci (Figure 4C). The array is most centrally localized while the major array-associated protein (53BP1 or BRCA1) associates with the array but extends beyond it. The minor component (53BP1 or BRCA1) shows a maximum that is well outside the position of the array, consistent with the more peripheral localization observed in images. The radial distribution profiles also reveal that these compartments are larger in the 53BP1-dominant foci versus the BRCA1-dominant foci. We confirmed the presence of DSBs using phosphorylated histone H2AX as a marker (Figure 4B). Thus, the array system behaves similarly to the IR-induced breaks and is suitable for more careful analysis of the relationships between these proteins and the break site.

### The Location of the Damaged DNA Relative to DSB-Associated Nanocompartments

**FIGURE 5:**
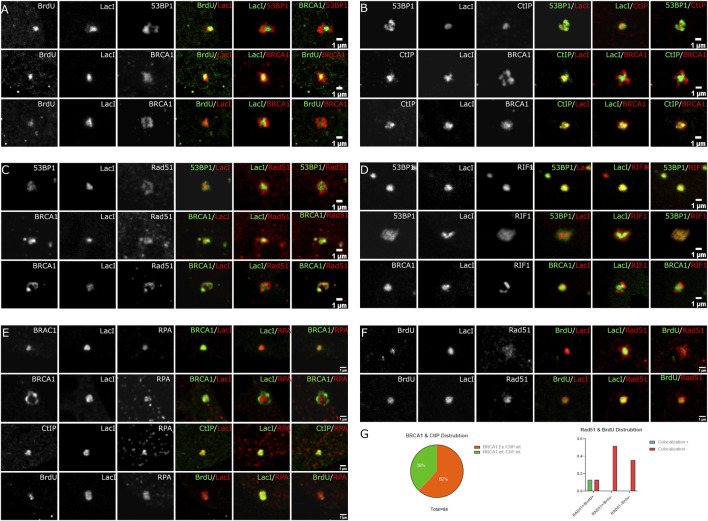
Spatial localization of BrdU labeling, CtIP, RAD51, Rif1, and RPA relative to LacI and the DSB compartment. U2OS 265 Fok1 induced DSB and fixed 1 h post-treatment and stained with antibodies recognizing 53BP1, BRCA1, BrdU, CtIP, Rad51, Rif1, and RPA. For the BrdU experiment, cells were labeled with BrdU for 18 h prior to treatment with Shield1 and 4-OHT. **(A)** BrdU distribution is compared to LacI. **(B)** CtIP localization is compared to LacI. Recruitment of Rad51 versus 53BP1 and BRCA1 relative to LacI **(C)** and Rif1 recruitment relative to 53BP1, BRCA1, and LacI **(D)**. **(E)** Localization of RPA compared with BRCA1, CtIP, and BrdU. **(F)** Different occupancy of Rad51 relative to end resection. **(G)** BRCA1 and CtIP distribution at DSB sites. BRCA1 exterior and CtIP interior versus BRCA1 interior and CtIP interior localizing to LacI. Rad51 and BrdU colocalization at the site of DNA break. The scale bar represents 1 µm.

The ability to directly detect the location of the DSB using the Lac repressor fusion protein allowed us to further assess spatial relationships relative to the break site. First, we addressed the location of DNA end resection. Cells were labeled with BrdU, and then the nuclease expression was induced. One hour later, cells were stained with an anti-BrdU antibody. In the absence of DNA denaturation, this detects only ssDNA and enables the identification of regions of the genome undergoing resection during DSB repair. We found that the BrdU always localized within the array volume (Figure 5A). This argues against a separate ssDNA compartment formed at DNA damage sites. 53BP1 was found on the periphery of the array in BrdU-positive cells. In contrast, we could observe BrdU-positive cells where BRCA1 was surrounding the array as well as BrdU-positive cells where BRCA1 associates with the array. When BRCA1 colocalized with the array and the BrdU, BRCA1 appeared to occupy a larger volume encompassing part of the periphery of the array. This indicates that the resected single-stranded DNA does not form a separate compartment from the double-stranded DNA when undergoing end resection, but that there may be a larger regulatory microenvironment that surrounds the array. We concluded that the ssDNA occupies a similar spatial space as the Lac repressor bound to the LacI repeats.

We next determined the location of CtIP, which is associated with BRCA1 and promotes the initiation of DNA end resection. CtIP was exclusively found within the volume of the array (Figure 5B). Notably, there remained two categories of BRCA1 distribution. We found that 32/84 recruit BRCA1 and CtIP where BRCA1 colocalized with CtIP on the array, while 52/84 exhibit BRCA1 on the periphery despite CtIP association with the array. Similar to the observations with BrdU, CtIP appears confined to the array while BRCA1 can extend beyond the array volume (Figure 4B). The CtIP localization is consistent with the BrdU labeling of single-stranded DNA and suggests that DNA end resection takes place directly on the DNA without spatial reorganization. It is also consistent with distinct BRCA1 complexes accumulating at DSBs and the BRCA1-independent activity of CtIP (Reczek et al., 2013; Polato et al., 2014).

We next assessed the relationship between Rad51 and the array (Figure 5C). Rad51 differed from CtIP and BrdU. We observed more than one organization of Rad51 relative to the array site. Rad51 could be associated predominantly with the periphery of the array or partially overlapping the array. We did not observe complete localization within the array volume, unlike BrdU and CtIP. This indicates that Rad51 is accumulating beyond the regions containing single-stranded DNA. It is unclear whether it is forming filaments in these regions.

Finally, we examined the 53BP1-associated inhibitor of DNA end resection, RIF1 (Figure 5D). RIF1 behaved as expected. In cells where 53BP1 encompasses the array volume, RIF1 also colocalized to the same volume. In cells where 53BP1 is associated with the peripheral regions of the array and excluded from the volume containing the array, RIF1 is also excluded from the array volume. When compared with BRCA1, like 53BP1, RIF1 localizes in a complementary rather than overlapping volume. This suggests that RIF1 localization is exclusively defined by 53BP1, consistent with their complex formation (Zimmermann et al., 2013).

### There Are at Least Three Subcompartments in DSB-Associated Foci

**FIGURE 6:**
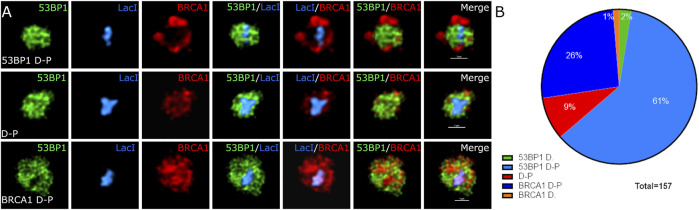
The site of DNA damage and relative proteins occupy specific spatially resolved sites. U2OS 265 cells were treated with 4-OHT and Shield1 to allow the translocation of mCherry–Fok1–Lac repressor to induce the DSB. Antibodies targeting 53BP1 and BRCA1 were used to determine recruitment to the DSB. **(A)** BRCA1 in green, 53BP1 in red, and Lac repressor in blue. Lightning—adaptive deconvolution (Leica)—was used to improve the resolution and image quality. **(B)** The proportion of cells in 53BP1-dominant cell (53BP1 D), 53BP1-dominant double-positive cell (53BP1 D-P), 53BP1–BRCA1-positive cell (D-P), BRCA1-dominant double-positive cell (BRCA1 D-P), and BRCA1-dominant cell (BRCA1 D) is also shown. The images were further magnified 400% using interpolation. The scale bar represents 1 µm.

The different distributions of 53BP1, BRCA1, single-stranded DNA, effector proteins, and the site of the DNA targeted with DSBs suggested that there may be more than two compartments associated with DNA DSB repair foci. To assess this further, we used deconvolution of laser scanning confocal images to improve resolution. Figure 6 shows BRCA1 (red), 53BP1 (green), and the Lac repressor–Fok1 fusion protein bound to the array (blue). BRCA1 and 53BP1 occupy distinct regions of the compartment independent of which is more centrally located. While the centrally located protein overlaps with the volume of the array, the protein typically does not completely occupy the same volume as the array and extends beyond the array volume. This suggests that there are at least three subcompartments within the focus including the damaged DNA, represented by the array location, the primary responding pathway, occupying the array-proximal volume, and the competing pathway factors, displaced to the outer volume of the compartment.

## Discussion

In this study, we investigated the variation in the organization and content of 53BP1 and BRCA1 in asynchronous cell populations upon DSB formation and demonstrated that there are sufficient similarities and differences to enable classification based on phenotype. When doing so, we find similar results to cell cycle-dependent studies demonstrating a gradual loss of 53BP1 foci during S-phase progression (Michelena et al., 2021). 53BP1 is a chromatin-binding protein that recognizes histone H4K20 mono/dimethylation through its Tudor domain and RNF168-mediated H2A K13/K15 ubiquitination through its UDR domain (Pei et al., 2011; Fradet-Turcotte et al., 2013). Inhibition of 53BP1 binding to chromatin may occur through epigenetic changes, including inhibitory histone acetylations and/or replication-dependent dilution of H4K20 methylation. During the S/G2 phase, Tip60 can acetylate H2A K13/15, which inhibits RNF168-mediated ubiquitination, and hMOF can acetylate H4K16, which inhibits the binding of 53BP1 to H4K20 methylation (Taipale et al., 2005; Mattiroli et al., 2012; Jacquet et al., 2016). Since histone H4 is deposited in an unmethylated form, dilution of the H4K20 methylation required for 53BP1 binding occurs during S phase. This is also correlated with the loss of 53BP1 binding (Saredi et al., 2016; Simonetta et al., 2018). The persistence of 53BP1 on newly replicated DNA early in S phase suggests that these replication-associated epigenetic changes are not sufficient to prevent 53BP1-rich focus assembly. Rather, our results suggest that BRCA1 nuclear concentration likely also plays a role. BRCA1 is cell cycle regulated in its expression (Jin et al., 1997; Choudhury et al., 2004). Consistent with this, we found that cells that assembled 53BP1-dominated foci split into two populations based on the amount of BRCA1 expression during G1. During S-phase progression, cells increasingly show BRCA1-rich foci as BRCA1 nuclear concentrations increase. The BARD1–BRCA1 complex can recognize H4K20me0 and H2A K15 ubiquitination to promote HR in S/G2 phase to preferentially bind newly replicated chromatin at the expense of 53BP1 (Saredi et al., 2016; Nakamura et al., 2019; Becker et al., 2021). The combination of increased expression and increased affinity of the BRCA1–BARD1 complex may be critical to mediate this reduction in 53BP1 occupancy. This is consistent with the observation that 53BP1 binding to S-phase damage sites is increased upon BRCA1 knockdown (Chapman et al., 2013; Escribano-Díaz et al., 2013; Michelena et al., 2021). Overall, the results are most consistent with an active competition based on the relative affinities of 53BP1 versus BRCA1 for the different epigenetic states pre- and postreplication, but argue that these modifications bias, rather than dictate, the outcome of this competition.

Our principal objective in this study was to understand protein organization within the DSB-associated compartment. For this purpose, we employed an integrated array system where we could induce a targeted DSB and, most importantly, know the location of the DNA containing the DSB. This allowed us to evaluate DSB compartment assembly in relation to the damage site rather than define localization relative to other DNA damage response proteins. Typically, phosphorylated histone H2AX is used to identify the site of a DNA DSB. However, phosphorylated histone H2AX is typically excluded from the site of the break (Arnould et al., 2021). A second advantage of the system is that the array is sufficiently large that it is easily identified, and subcompartments are characterized without a requirement for super-resolution microscopy approaches. We had previously used electron spectroscopic imaging, an analytical transmission electron microscopy capable of identifying DNA and RNA based on its abundance of phosphorus, to demonstrate that chromatin is compartmentalized within DSB repair foci (Strickfaden et al., 2015). Our observation that chromatin was enriched on the exterior of foci with 53BP1-rich centers suggested that this could be a site of sequestration away from the repair site. By knowing the location of the DNA DSB, we can now rule this out. The electron microscopy results, however, suggest that chromatin density is much lower in the interior of the focus. While there is evidence from super-resolution microscopy experiments that BRCA1 centralizes and displaces 53BP1 to the periphery (Chapman et al., 2012), we observed that the opposite organization, with centralized 53BP1 and peripherally located BRCA1, also exists within populations of asynchronous cells. While this could reflect an early stage in a process of displacement, this appears unlikely given that this organization correlates with overall DNA content, which further correlates with BRCA1 abundance. Hence, we favor a model where both can bind, that their affinity is modulated by replication-dependent epigenetic changes in the chromatin template, and influenced by the relative expression of each protein. Similar conclusions were recently reached by Michelena et al. (2021).

The central region of the compartment containing the array was determined to be the site of DNA end resection and single-stranded DNA accumulation. BrdU labeling revealed that the single-stranded DNA co-occupied the same volume as the array. If liquid–liquid phase separation occurs within DSB compartments, the separation of single-stranded DNA into a separate compartment could conceivably take place, so this is important to establish. CtIP has also been shown to colocalize with BRCA1 by structured illumination microscopy (Chapman et al., 2012), consistent with our results obtained on the array; however, we found instances where the two signals appeared independent. This could reflect an abundance of the BRCA1 A complex (Kim et al., 2007; Sobhian et al., 2007; Wang et al., 2007) relative to BRCA1/CtIP complexes, or it could reflect BRCA1-independent CtIP localization (Sobhian et al., 2007; Polato et al., 2014). In these instances, BRCA1 was positioned external to CtIP. Rad51, unlike CtIP, tended to localize on the immediate periphery of the array as well as associate with it. It may be that the 1-h time point precedes the assembly of Rad51 into filaments and that it accumulates prior to assembly. As expected, the 53BP1-associated protein RIF1 colocalized with 53BP1 and could exist either on the array or, more commonly, displaced from the region containing the array.

Triple-labeling experiments revealed that the array occupies a distinct space that only partially overlaps with BRCA1 or 53BP1-rich domains. It is notable that BRCA1 and 53BP1, when localized to the array, also encompass it, while CtIP or BrdU (ssDNA) are constrained within the volume of the array. It was also surprising to observe Rad51 surrounding the array rather than confined to the array volume. It may be that this reflects an early point in the assembly onto ssDNA. This suggests the existence of at least three microenvironments within the DNA damage focus. Ultrastructural studies would assist in the interpretation of DSB repair focus organization.

## Data Availability

The raw data supporting the conclusions of this article will be made available by the authors, without undue reservation.

## References

[B1] Akhtar A. Becker P. B. (2000). Activation of Transcription through Histone H4 Acetylation by MOF, an Acetyltransferase Essential for Dosage Compensation in Drosophila. Mol. Cell 5 (2), 367–375. 10.1016/s1097-2765(00)80431-1 PubMed Abstract | 10.1016/s1097-2765(00)80431-1 | Google Scholar 10882077

[B2] Altmeyer M. Neelsen K. J. Teloni F. Pozdnyakova I. Pellegrino S. Grøfte M. (2015). Liquid Demixing of Intrinsically Disordered Proteins Is Seeded by poly(ADP-Ribose). Nat. Commun. 6. 10.1038/ncomms9088 10.1038/ncomms9088 | Google Scholar PMC4560800 26286827

[B3] Arnould C. Rocher V. Finoux A.-L. Clouaire T. Li K. Zhou F. (2021). Loop Extrusion as a Mechanism for Formation of DNA Damage Repair Foci. Nature 590, 660–665. 10.1038/s41586-021-03193-z PubMed Abstract | 10.1038/s41586-021-03193-z | Google Scholar 33597753 PMC7116834

[B4] Aymard F. Bugler B. Schmidt C. K. Guillou E. Caron P. Briois S. (2014). Transcriptionally Active Chromatin Recruits Homologous Recombination at DNA Double-Strand Breaks. Nat. Struct. Mol. Biol. 21, 366–374. 10.1038/nsmb.2796 PubMed Abstract | 10.1038/nsmb.2796 | Google Scholar 24658350 PMC4300393

[B5] Aymard F. Legube G. (2016). A TAD Closer to ATM. Mol. Cell. Oncol. 3, e1134411. 10.1080/23723556.2015.1134411 PubMed Abstract | 10.1080/23723556.2015.1134411 | Google Scholar 27314089 PMC4909466

[B6] Becker J. R. Clifford G. Bonnet C. Groth A. Wilson M. D. Chapman J. R. (2021). BARD1 Reads H2A Lysine 15 Ubiquitination to Direct Homologous Recombination. Nature 596, 433–437. 10.1038/s41586-021-03776-w PubMed Abstract | 10.1038/s41586-021-03776-w | Google Scholar 34321663 PMC7619424

[B7] Bekker-Jensen S. Lukas C. Melander F. Bartek J. Lukas J. (2005). Dynamic Assembly and Sustained Retention of 53BP1 at the Sites of DNA Damage Are Controlled by Mdc1/NFBD1. J. Cell Biol. 170, 201–211. 10.1083/jcb.200503043 PubMed Abstract | 10.1083/jcb.200503043 | Google Scholar 16009723 PMC2171401

[B8] Bekker-Jensen S. Mailand N. (2010). Assembly and Function of DNA Double-Strand Break Repair Foci in Mammalian Cells. DNA Repair 9, 1219–1228. 10.1016/j.dnarep.2010.09.010 PubMed Abstract | 10.1016/j.dnarep.2010.09.010 | Google Scholar 21035408

[B9] Bouwman P. Aly A. Escandell J. M. Pieterse M. Bartkova J. van der Gulden H. (2010). 53BP1 Loss Rescues BRCA1 Deficiency and Is Associated with Triple-Negative and BRCA-Mutated Breast Cancers. Nat. Struct. Mol. Biol. 17, 688–695. 10.1038/nsmb.1831 PubMed Abstract | 10.1038/nsmb.1831 | Google Scholar 20453858 PMC2912507

[B10] Bunting S. F. Callén E. Wong N. Chen H.-T. Polato F. Gunn A. (2010). 53BP1 Inhibits Homologous Recombination in Brca1-Deficient Cells by Blocking Resection of DNA Breaks. Cell 141, 243–254. 10.1016/j.cell.2010.03.012 PubMed Abstract | 10.1016/j.cell.2010.03.012 | Google Scholar 20362325 PMC2857570

[B11] Caron P. Aymard F. Iacovoni J. S. Briois S. Canitrot Y. Bugler B. (2012). Cohesin Protects Genes against γH2AX Induced by DNA Double-Strand Breaks. PLoS Genet. 8, e1002460. 10.1371/journal.pgen.1002460 PubMed Abstract | 10.1371/journal.pgen.1002460 | Google Scholar 22275873 PMC3261922

[B12] Chapman J. R. Barral P. Vannier J.-B. Borel V. Steger M. Tomas-Loba A. (2013). RIF1 Is Essential for 53BP1-dependent Nonhomologous End Joining and Suppression of DNA Double-Strand Break Resection. Mol. Cell 49, 858–871. 10.1016/j.molcel.2013.01.002 PubMed Abstract | 10.1016/j.molcel.2013.01.002 | Google Scholar 23333305 PMC3594748

[B13] Chapman J. R. Sossick A. J. Boulton S. J. Jackson S. P. (2012). BRCA1-associated Exclusion of 53BP1 from DNA Damage Sites Underlies Temporal Control of DNA Repair. J. Cell Sci. 125, 3529–3534. 10.1242/jcs.105353 PubMed Abstract | 10.1242/jcs.105353 | Google Scholar 22553214 PMC3445322

[B14] Chen L. Nievera C. J. Lee A. Y.-L. Wu X. (2008). Cell Cycle-dependent Complex Formation of BRCA1·CtIP·MRN Is Important for DNA Double-Strand Break Repair. J. Biol. Chem. 283, 7713–7720. 10.1074/jbc.M710245200 PubMed Abstract | 10.1074/jbc.M710245200 | Google Scholar 18171670

[B15] Choudhury A. D. Xu H. Baer R. (2004). Ubiquitination and Proteasomal Degradation of the BRCA1 Tumor Suppressor Is Regulated during Cell Cycle Progression. J. Biol. Chem. 279, 33909–33918. 10.1074/jbc.M403646200 PubMed Abstract | 10.1074/jbc.M403646200 | Google Scholar 15166217

[B16] Doil C. Mailand N. Bekker-Jensen S. Menard P. Larsen D. H. Pepperkok R. (2009). RNF168 Binds and Amplifies Ubiquitin Conjugates on Damaged Chromosomes to Allow Accumulation of Repair Proteins. Cell 136, 435–446. 10.1016/j.cell.2008.12.041 PubMed Abstract | 10.1016/j.cell.2008.12.041 | Google Scholar 19203579

[B17] Escribano-Díaz C. Orthwein A. Fradet-Turcotte A. Xing M. Young J. T. F. Tkáč J. (2013). A Cell Cycle-dependent Regulatory Circuit Composed of 53BP1-RIF1 and BRCA1-CtIP Controls DNA Repair Pathway Choice. Mol. Cell 49, 872–883. 10.1016/j.molcel.2013.01.001 PubMed Abstract | 10.1016/j.molcel.2013.01.001 | Google Scholar 23333306

[B18] Feng L. Li N. Li Y. Wang J. Gao M. Wang W. (2015). Cell Cycle-dependent Inhibition of 53BP1 Signaling by BRCA1. Cell Discov. 1. 10.1038/celldisc.2015.19 PubMed Abstract | 10.1038/celldisc.2015.19 | Google Scholar PMC4860855 27462418

[B19] Fradet-Turcotte A. Canny M. D. Escribano-Díaz C. Orthwein A. Leung C. C. Y. Huang H. (2013). 53BP1 Is a Reader of the DNA-Damage-Induced H2A Lys 15 Ubiquitin Mark. Nature 499, 50–54. 10.1038/nature12318 PubMed Abstract | 10.1038/nature12318 | Google Scholar 23760478 PMC3955401

[B20] Gong Z. Cho Y.-W. Kim J.-E. Ge K. Chen J. (2009). Accumulation of Pax2 Transactivation Domain Interaction Protein (PTIP) at Sites of DNA Breaks via RNF8-dependent Pathway Is Required for Cell Survival after DNA Damage. J. Biol. Chem. 284, 7284–7293. 10.1074/jbc.M809158200 PubMed Abstract | 10.1074/jbc.M809158200 | Google Scholar 19124460 PMC2652327

[B21] Guo Q. Shi X. Wang X. (2021). RNA and Liquid-Liquid Phase Separation. Non-coding RNA Res. 6, 92–99. 10.1016/j.ncrna.2021.04.003 10.1016/j.ncrna.2021.04.003 | Google Scholar PMC8111091 33997539

[B22] Huen M. S. Y. Grant R. Manke I. Minn K. Yu X. Yaffe M. B. (2007). RNF8 Transduces the DNA-Damage Signal via Histone Ubiquitylation and Checkpoint Protein Assembly. Cell 131, 901–914. 10.1016/j.cell.2007.09.041 PubMed Abstract | 10.1016/j.cell.2007.09.041 | Google Scholar 18001825 PMC2149842

[B23] Hustedt N. Durocher D. (2017). The Control of DNA Repair by the Cell Cycle. Nat. Cell Biol. 19, 1–9. 10.1038/ncb3452 10.1038/ncb3452 | Google Scholar 28008184

[B24] Iacovoni J. S. Caron P. Lassadi I. Nicolas E. Massip L. Trouche D. (2010). High-resolution Profiling of γH2AX Around DNA Double Strand Breaks in the Mammalian Genome. Embo J. 29, 1446–1457. 10.1038/emboj.2010.38 PubMed Abstract | 10.1038/emboj.2010.38 | Google Scholar 20360682 PMC2868577

[B25] Jackson S. P. (2002). Sensing and Repairing DNA Double-Strand Breaks. Carcinogenesis 23, 687–696. 10.1043/carcin/23.5.687 PubMed Abstract | 10.1043/carcin/23.5.687 | Google Scholar 12016139

[B26] Jacquet K. Fradet-Turcotte A. Avvakumov N. Lambert J.-P. Roques C. Pandita R. K. (2016). The TIP60 Complex Regulates Bivalent Chromatin Recognition by 53BP1 through Direct H4K20me Binding and H2AK15 Acetylation. Mol. Cell 62, 409–421. 10.1016/j.molcel.2016.03.031 PubMed Abstract | 10.1016/j.molcel.2016.03.031 | Google Scholar 27153538 PMC4887106

[B27] Jin Y. Xu X. L. Yang M.-C. W. Wei F. Ayi T.-C. Bowcock A. M. (1997). Cell Cycle-dependent Colocalization of BARD1 and BRCA1 Proteins in Discrete Nuclear Domains. Proc. Natl. Acad. Sci. 94 (22), 12075–12080. 10.1073/pnas.94.22.12075 PubMed Abstract | 10.1073/pnas.94.22.12075 | Google Scholar 9342365 PMC23707

[B28] Kilic S. Lezaja A. Gatti M. Bianco E. Michelena J. Imhof R. (2019). Phase Separation of 53 BP 1 Determines Liquid‐like Behavior of DNA Repair Compartments. Embo J. 38. 10.15252/embj.2018101379 10.15252/embj.2018101379 | Google Scholar PMC6694294 31267591

[B29] Kim H. Chen J. Yu X. (2007). Ubiquitin-Binding Protein RAP80 Mediates BRCA1-dependent DNA Damage Response. Science 316, 1202–1205. 10.1126/science.1139621 PubMed Abstract | 10.1126/science.1139621 | Google Scholar 17525342

[B30] Kolas N. K. Chapman J. R. Nakada S. Ylanko J. Chahwan R. Sweeney F. D. (2007). Orchestration of the DNA-Damage Response by the RNF8 Ubiquitin Ligase. Science 318 (5856), 1637–1640. 10.1126/science.1150034 PubMed Abstract | 10.1126/science.1150034 | Google Scholar 18006705 PMC2430610

[B31] Krais J. J. Wang Y. Patel P. Basu J. Bernhardy A. J. Johnson N. (2021). RNF168-mediated Localization of BARD1 Recruits the BRCA1-PALB2 Complex to DNA Damage. Nat. Commun. 12. 10.1038/s41467-021-25346-4 10.1038/s41467-021-25346-4 | Google Scholar PMC8373961 34408138

[B32] Lavin M. F. (2004). The Mre11 Complex and ATM: A Two-Way Functional Interaction in Recognising and Signaling DNA Double Strand Breaks. DNA Repair 3, 1515–1520. 10.1016/j.dnarep.2004.07.001 PubMed Abstract | 10.1016/j.dnarep.2004.07.001 | Google Scholar 15380107

[B33] Lee J.-H. Paull T. T. (2005). ATM Activation by DNA Double-Strand Breaks through the Mre11-Rad50-Nbs1 Complex. Science 308 (5721), 551–554. 10.1126/science.1108297 PubMed Abstract | 10.1126/science.1108297 | Google Scholar 15790808

[B34] Li X. Corsa C. A. S. Pan P. W. Wu L. Ferguson D. Yu X. (2010). MOF and H4 K16 Acetylation Play Important Roles in DNA Damage Repair by Modulating Recruitment of DNA Damage Repair Protein Mdc1. Mol. Cell Biol. 30, 5335–5347. 10.1128/mcb.00350-10 PubMed Abstract | 10.1128/mcb.00350-10 | Google Scholar 20837706 PMC2976376

[B35] Mailand N. Bekker-Jensen S. Faustrup H. Melander F. Bartek J. Lukas C. (2007). RNF8 Ubiquitylates Histones at DNA Double-Strand Breaks and Promotes Assembly of Repair Proteins. Cell 131, 887–900. 10.1016/j.cell.2007.09.040 PubMed Abstract | 10.1016/j.cell.2007.09.040 | Google Scholar 18001824

[B36] Mattiroli F. Vissers J. H. A. van Dijk W. J. Ikpa P. Citterio E. Vermeulen W. (2012). RNF168 Ubiquitinates K13-15 on H2A/H2AX to Drive DNA Damage Signaling. Cell 150, 1182–1195. 10.1016/j.cell.2012.08.005 PubMed Abstract | 10.1016/j.cell.2012.08.005 | Google Scholar 22980979

[B37] Michelena J. Pellegrino S. Spegg V. Altmeyer M. (2021). Replicated Chromatin Curtails 53BP1 Recruitment in BRCA1-Proficient and BRCA1-Deficient Cells. Life Sci. Alliance 4, e202101023–10. 10.26508/LSA.202101023 PubMed Abstract | 10.26508/LSA.202101023 | Google Scholar 33811064 PMC8046418

[B38] Mok M. T. S. Henderson B. R. (2012). Three-dimensional Imaging Reveals the Spatial Separation of γH2AX-MDC1-53BP1 and RNF8-Rnf168-BRCA1-A Complexes at Ionizing Radiation-Induced Foci. Radiotherapy Oncol. 103, 415–420. 10.1016/j.radonc.2012.04.009 PubMed Abstract | 10.1016/j.radonc.2012.04.009 | Google Scholar 22633816

[B39] Nakamura K. Saredi G. Becker J. R. Foster B. M. Nguyen N. v. Beyer T. E. (2019). H4K20me0 Recognition by BRCA1-BARD1 Directs Homologous Recombination to Sister Chromatids. Nat. Cell Biol. 21, 311–318. 10.1038/s41556-019-0282-9 PubMed Abstract | 10.1038/s41556-019-0282-9 | Google Scholar 30804502 PMC6420097

[B40] Nesterov S. v. Ilyinsky N. S. Uversky V. N. (2021). Liquid-liquid Phase Separation as a Common Organizing Principle of Intracellular Space and Biomembranes Providing Dynamic Adaptive Responses. Biochimica Biophysica Acta (BBA) - Mol. Cell Res. 1868, 119102. 10.1016/j.bbamcr.2021.119102 10.1016/j.bbamcr.2021.119102 | Google Scholar 34293345

[B41] Pei H. Zhang L. Luo K. Qin Y. Chesi M. Fei F. (2011). MMSET Regulates Histone H4K20 Methylation and 53BP1 Accumulation at DNA Damage Sites. Nature 470, 124–128. 10.1038/nature09658 PubMed Abstract | 10.1038/nature09658 | Google Scholar 21293379 PMC3064261

[B42] Pessina F. Giavazzi F. Yin Y. Gioia U. Vitelli V. Galbiati A. (2019). Functional Transcription Promoters at DNA Double-Strand Breaks Mediate RNA-Driven Phase Separation of Damage-Response Factors. Nat. Cell Biol. 21, 1286–1299. 10.1038/s41556-019-0392-4 PubMed Abstract | 10.1038/s41556-019-0392-4 | Google Scholar 31570834 PMC6859070

[B43] Polato F. Callen E. Wong N. Faryabi R. Bunting S. Chen H.-T. (2014). CtIP-mediated Resection Is Essential for Viability and Can Operate Independently of BRCA1. J. Exp. Med. 211, 1027–1036. 10.1084/jem.20131939 PubMed Abstract | 10.1084/jem.20131939 | Google Scholar 24842372 PMC4042650

[B44] Rack J. G. M. Liu Q. Zorzini V. Voorneveld J. Ariza A. Honarmand Ebrahimi K. (2021). Mechanistic Insights into the Three Steps of poly(ADP-Ribosylation) Reversal. Nat. Commun. 12. 10.1038/s41467-021-24723-3 10.1038/s41467-021-24723-3 | Google Scholar PMC8319183 34321462

[B45] Razin S. v. Gavrilov A. A. (2020). The Role of Liquid-Liquid Phase Separation in the Compartmentalization of Cell Nucleus and Spatial Genome Organization. Biochem. Mosc. 85, 643–650. 10.1134/S0006297920060012 PubMed Abstract | 10.1134/S0006297920060012 | Google Scholar 32586227

[B46] Reczek C. R. Szabolcs M. Stark J. M. Ludwig T. Baer R. (2013). The Interaction between CtIP and BRCA1 Is Not Essential for Resection-Mediated DNA Repair or Tumor Suppression. J. Cell Biol. 201, 693–707. 10.1083/jcb.201302145 PubMed Abstract | 10.1083/jcb.201302145 | Google Scholar 23712259 PMC3664708

[B47] Reindl J. Girst S. Walsh D. W. M. Greubel C. Schwarz B. Siebenwirth C. (2017). Chromatin Organization Revealed by Nanostructure of Irradiation Induced γH2AX, 53BP1 and Rad51 Foci. Sci. Rep. 7. 10.1038/srep40616 PubMed Abstract | 10.1038/srep40616 | Google Scholar PMC5240115 28094292

[B48] Reindl J. Kundrat P. Girst S. Sammer M. Schwarz B. Dollinger G. (2022). Dosimetry of Heavy Ion Exposure to Human Cells Using Nanoscopic Imaging of Double Strand Break Repair Protein Clusters. Sci. Rep. 12. 10.1038/s41598-022-05413-6 10.1038/s41598-022-05413-6 | Google Scholar PMC8789836 35079078

[B49] Saredi G. Huang H. Hammond C. M. Alabert C. Bekker-Jensen S. Forne I. (2016). H4K20me0 Marks Post-replicative Chromatin and Recruits the TONSL-Mms22l DNA Repair Complex. Nature 534, 714–718. 10.1038/nature18312 PubMed Abstract | 10.1038/nature18312 | Google Scholar 27338793 PMC4939875

[B50] Schwarz B. Friedl A. A. Girst S. Dollinger G. Reindl J. (2019). Nanoscopic Analysis of 53BP1, BRCA1 and Rad51 Reveals New Insights in Temporal Progression of DNA-Repair and Pathway Choice. Mutat. Research/Fundamental Mol. Mech. Mutagen. 816-818, 111675–111818. 10.1016/j.mrfmmm.2019.111675 PubMed Abstract | 10.1016/j.mrfmmm.2019.111675 | Google Scholar 31302572

[B51] Simonetta M. de Krijger I. Serrat J. Moatti N. Fortunato D. Hoekman L. (2018). H4K20me2 Distinguishes Pre-replicative from Post-replicative Chromatin to Appropriately Direct DNA Repair Pathway Choice by 53BP1-RIF1-Mad2l2. Cell Cycle 17, 124–136. 10.1080/15384101.2017.1404210 PubMed Abstract | 10.1080/15384101.2017.1404210 | Google Scholar 29160738 PMC5815438

[B52] Sobhian B. Shao G. Lilli D. R. Culhane A. C. Moreau L. A. Xia B. (2007). RAP80 Targets BRCA1 to Specific Ubiquitin Structures at DNA Damage Sites. Science 316, 1198–1202. 10.1126/science.1139516 PubMed Abstract | 10.1126/science.1139516 | Google Scholar 17525341 PMC2706583

[B61] Stewart G. S. Panier S. Townsend K. Al-Hakim A. K. Kolas N. K. Miller E. S. (2009). The RIDDLE Syndrome Protein Mediates a Ubiquitin-Dependent Signaling Cascade at Sites of DNA Damage. Cell 136, 420–434. 10.1016/j.cell.2008.12.042 PubMed Abstract | 10.1016/j.cell.2008.12.042 | Google Scholar 19203578

[B53] Strickfaden H. Xu Z. Z. Hendzel M. J. (2015). Visualization of miniSOG Tagged DNA Repair Proteins in Combination with Electron Spectroscopic Imaging (ESI). JoVE 103. 10.3791/52893 PubMed Abstract | 10.3791/52893 | Google Scholar PMC4692621 26436332

[B54] Stucki M. Jackson S. P. (2004). MDC1/NFBD1: A Key Regulator of the DNA Damage Response in Higher Eukaryotes. DNA Repair 3, 953–957. 10.1016/j.dnarep.2004.03.007 PubMed Abstract | 10.1016/j.dnarep.2004.03.007 | Google Scholar 15279781

[B55] Swift M. L. Beishline K. Flashner S. Azizkhan-Clifford J. (2021). DSB Repair Pathway Choice Is Regulated by Recruitment of 53BP1 through Cell Cycle-dependent Regulation of Sp1. Cell Rep. 34, 108840. 10.1016/j.celrep.2021.108840 PubMed Abstract | 10.1016/j.celrep.2021.108840 | Google Scholar 33730584

[B56] Taipale M. Rea S. Richter K. Vilar A. Lichter P. Imhof A. (2005). hMOF Histone Acetyltransferase Is Required for Histone H4 Lysine 16 Acetylation in Mammalian Cells. Mol. Cell Biol. 25, 6798–6810. 10.1128/mcb.25.15.6798-6810.2005 PubMed Abstract | 10.1128/mcb.25.15.6798-6810.2005 | Google Scholar 16024812 PMC1190338

[B57] Tang J. Cho N. W. Cui G. Manion E. M. Shanbhag N. M. Botuyan M. V. (2013). Acetylation Limits 53BP1 Association with Damaged Chromatin to Promote Homologous Recombination. Nat. Struct. Mol. Biol. 20, 317–325. 10.1038/nsmb.2499 PubMed Abstract | 10.1038/nsmb.2499 | Google Scholar 23377543 PMC3594358

[B58] Wang B. Matsuoka S. Ballif B. A. Zhang D. Smogorzewska A. Gygi S. P. (2007). Abraxas and RAP80 Form a BRCA1 Protein Complex Required for the DNA Damage Response. Science 316, 1194–1198. 10.1126/science.1139476 PubMed Abstract | 10.1126/science.1139476 | Google Scholar 17525340 PMC3573690

[B59] Zhang L. Geng X. Wang F. Tang J. Ichida Y. Sharma A. (2022). 53BP1 Regulates Heterochromatin through Liquid Phase Separation. Nat. Commun. 13, 360. 10.1038/s41467-022-28019-y PubMed Abstract | 10.1038/s41467-022-28019-y | Google Scholar 35042897 PMC8766474

[B60] Zimmermann M. Lottersberger F. Buonomo S. B. Sfeir A. de Lange T. (2013). 53BP1 Regulates DSB Repair Using Rif1 to Control 5′ End Resection. Science 339, 700–704. 10.1126/science.1231573 PubMed Abstract | 10.1126/science.1231573 | Google Scholar 23306437 PMC3664841

